# Fucosylation in digestive inflammatory diseases and cancers: From mechanical studies to clinical translation

**DOI:** 10.1016/j.gendis.2025.101570

**Published:** 2025-02-22

**Authors:** Caihan Duan, Junhao Wu, Zhe Wang, Xiaohua Hou, Chaoqun Han

**Affiliations:** Department of Gastroenterology, Union Hospital, Tongji Medical College, Huazhong University of Science and Technology, Wuhan, Hubei 430000, China

**Keywords:** Cancer, Digestive system, Fucosylation, Fucosyltransferases, Inflammation

## Abstract

Fucosylation is a post-translational modification that attaches fucose to glycoproteins or glycolipids, thereby influencing their biological functions. Consequently, fucosylation proves indispensable for many biological processes, such as ligand–receptor interaction, cell adhesion, and signal transduction, holding critical clinical significance in the genesis and development of diseases. Recent studies further unveiled the clinical significance and molecular mechanism underlying the pathogenetic role of aberrant fucosylation. Herein, we summarize the effects of fucosylation in digestive system inflammatory diseases and cancers, primarily concentrating on the intestine, stomach, liver, and pancreas, from the aspects of the genetic risks of fucosyltransferase mutation, the roles of aberrant fucosylated glycans as diagnostic biomarkers, and the molecular mechanisms of fucosylation-related gene-induced disorders. Finally, we discuss therapeutic strategies targeting fucosylation by fucose or fucosylation inhibitors. We aim to elaborate on the current understanding and provide novel insights into the role of fucosylation in digestive diseases, hoping to facilitate future studies and resolve clinical issues.

## Introduction

Glycosylation is an elaborate post-translational modification process, which is vital for the formation of protein biological activity.^1^ Fucosylation is one kind of glycosylation that attaches fucose to proteins and lipids under the regulation of fucosyltransferases (FUTs) in the endoplasmic reticulum and the Golgi apparatus.^2^ It has been demonstrated that fucosylation plays a crucial role in modulating biological processes such as intracellular trafficking, ligand–receptor interaction, cell adhesion, and signal transduction.^3^, ^4^, ^5^ Consequently, in recent years, an accumulation of research has focused on the mechanism by which fucosylation influences health and disease.

There are numerous fucosylated proteins in the digestive tract, ranging from mucins to cell membrane proteins, which are involved in the formation of the intestinal barrier. Since the digestive tract is continuously exposed to environmental antigens such as microbes, the cellular barrier and mucus barrier functions are essential for gut homeostasis.^6^ Therefore, the effects of fucosylation are particularly prominent in the digestive tract. Alterations in fucosylation were considered to be related to many digestive diseases such as inflammatory bowel disease, liver cirrhosis, chronic pancreatitis, and atrophic gastritis.^7^, ^8^, ^9^, ^10^ Some fucosylated proteins can also serve as biomarkers for the diagnosis and differential diagnosis of these diseases.^11^ Importantly, the effects of the same or different kinds of fucosylation and FUTs may vary or even opposite in different diseases. However, the overall understanding of the mechanism of aberrant fucosylation-induced digestive diseases remains insufficient.

In this review, we provide an overview of the current knowledge regarding the role of fucosylation in inflammatory diseases and cancers of the alimentary system, as well as the diagnostic biomarkers and probable underlying mechanisms. Additionally, we discuss the potential therapeutic strategies related to fucosylation such as l-fucose supplements and fucosylation inhibitors, which have shown significant effects in disease models.

## Biological process and functions of fucosylation

**Figure 1:**
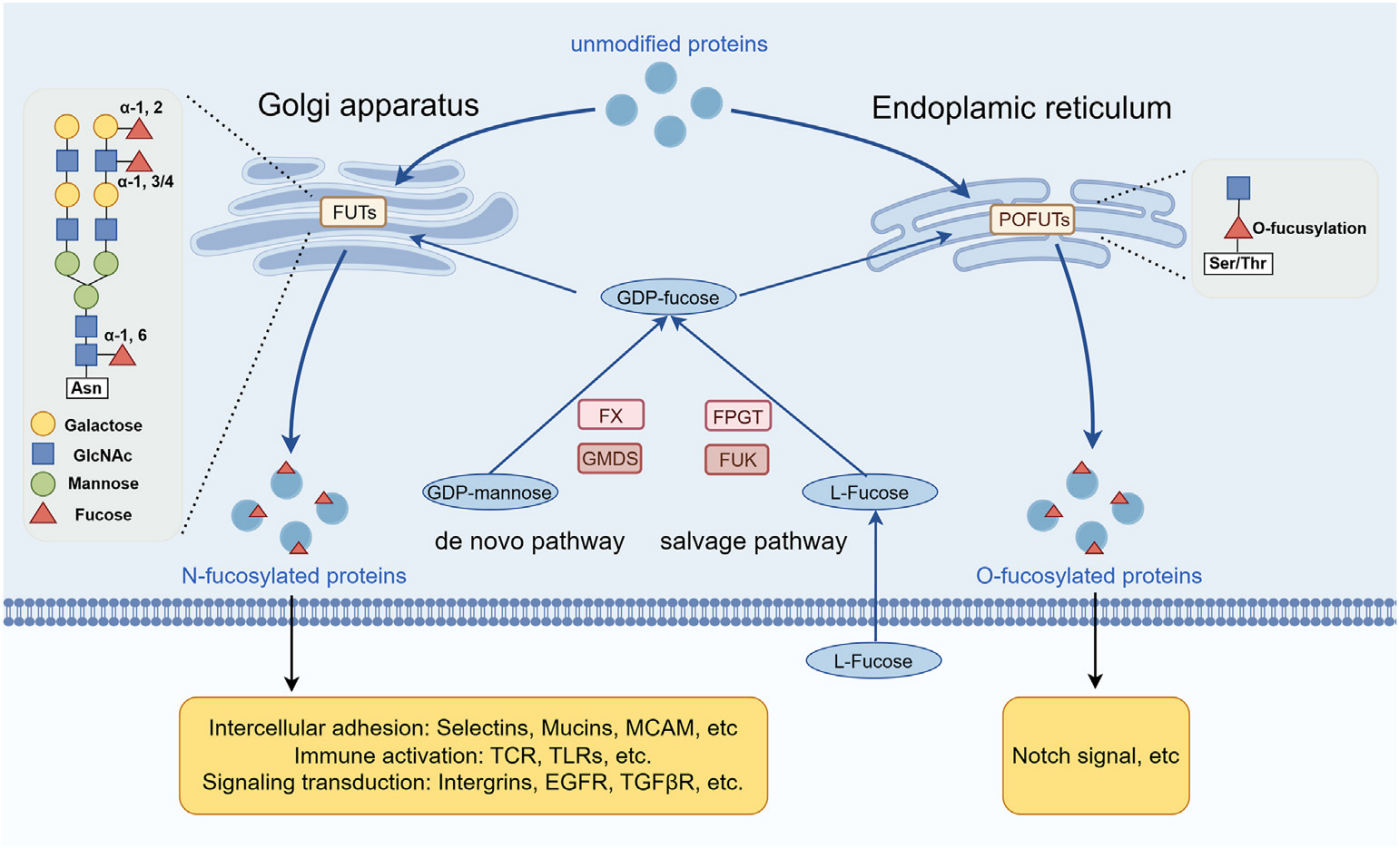
Biological process of fucosylation. The substrate of fucosylation, GDP-fucose, can be derived from the *de novo* and salvage pathway. In the *de novo* pathway, GDP-mannose is transformed to GDP-fucose catalyzed by FX and GMDS, while in the salvage pathway, GDP-fucose is derived from exogenous l-fucose under the control of FUK and FPGT. GDP-fucose is transport to Golgi apparatus for α1, 2, α1, 3/4, and α1, 6-fucosylation that catalyzed by FUTs, and to ER for O-fucosylation that catalyzed by POFUTs. GMDS, GDP-mannose 4,6-dehydratase; FX, GDP-fucose synthetic enzyme; FUK, fucose kinase; FPGT, fucose-1-phosphate guanylyltransferase; FUTs, fucosyltransferases; ER, endoplasmic reticulum; POFUTs, protein O-fucosyltransferases.

As shown in Figure 1, the substrate of fucosylation, guanosine diphosphate-fucose (GDP-fucose), can be derived from the *de novo* and salvage pathway. The *de novo* pathway predominates in the synthesis of GDP-fucose, which converts GDP-mannose to GDP-fucose and relies on GDP-mannose 4,6-dehydratase (GMDS) and GDP-fucose synthetic enzyme (FX, or tissue-specific transplantation antigen P35B, TSTA3).^12^^,^^13^ In the salvage pathway, free fucose is transformed to GDP-fucose mediated by fucose kinase and fucose-1-phosphate guanylyltransferase.^14^ In addition, exogenous supplementation of fucose could activate the salvage pathway when the *de novo* pathway is dysfunctional.^15^ The GDP-fucose is subsequently transported to the Golgi apparatus for FUT-catalyzed N-fucosylation (where fucose is linked to asparagine residues) or transported to endoplasmic reticulum for protein O-fucosyltransferase (POFUT)-catalyzed O-fucosylation (where fucose is linked to serine or threonine residues in epidermal growth factor (EGF)-like and thrombospondin type 1 repeat (TSR) domains).^16^^,^^17^ N-fucosylation encompasses α1, 2, α1, 3/4, and α1,6-fucosylation. Fucose is attached to the terminal of molecules in α1, 2 and α1,3/4-fucosylation, while α1,6-fucosylation (also known as core fucosylation) occurs at the innermost N-acetylglucosamine (GlcNAc) residue of N-glycans.

FUT1 and FUT2 catalyze α1, 2-fucosylation, which constitutes a major form of fucosylation in intestinal epithelial cells, functioning at the interface of host–microbe interaction.^18^ Significantly, functional FUT2 enables the secretion of histo-blood group antigens (HBGAs) in gastrointestinal mucosa by modifying the H antigens, and the mutation of FUT2 (non-secretors) gives rise to susceptibility to diseases such as enteric pathogen infections and inflammatory bowel disease. FUT3-7 and FUT9 catalyze α1, 3/4-fucosylation, also known as the Lewis type. The most representative effect of Lewis glycans on the cell surface is to serve as sites for *Helicobacter pylori* (*H. pylori*) infection in *H. pylori*-induced gastric cancer (GC). FUT8 catalyzes α1, 6-fucosylation, which plays a crucial role in gastrointestinal immunity since most immune molecules are core fucosylated glycoproteins.^19^ FUT10 and FUT11 are also α1, 3-fucosyltransferases that differ from classical α1, 3-fucosyltransferases as they do not attach fucose to short lactosaminyl acceptor substrates.^20^ POFUT1 and POFUT2 regulate the O-fucosylation, which is involved in the binding of molecules such as Notch and its ligands.^21^

The fucosylated glycans on the cell surface mainly participate in cell recognition and signaling transduction. i) Intercellular adhesion: on the one hand, fucosylated selectins are indispensable for the adhesion of leukocytes to endothelial cells.^4^ On the other hand, the fucosylated glycans on gastrointestinal epithelial cells are critical sites for colonization and infection of commensal microbes and pathogens and can be utilized by microbes.^22^ ii) Immune activation: the highly fucosylated T cell receptor and CD14/Toll-like receptor (TLR) complexes make fucosylation important for T cell activation, T cell-B cell interaction, and TLR-mediated microphage activation in gastrointestinal immune modulation.^23^ iii) Signaling transduction: many receptors on the cell surface are highly fucosylated, such as Notch, transforming growth factor beta (TGFβ) receptors, epidermal growth factor receptor (EGFR), and integrins. Therefore, aberrant fucosylation influences the activity of these receptors and the signaling transduction they mediate. Furthermore, intracellular fucosylated proteins often regulate organelle function and relevant biological processes. For example, the fucosylation of lysosome-associated membrane proteins was related to lysosome location and autophagy process.^24^

The disorder of fucosylation and fucosylation-related genes was demonstrated to have an impact on the development of inflammation and cancers in the digestive system, and we will elaborate on them from the perspective of clinical association, biomarkers, molecular mechanisms, and potential therapy approaches of each disease in the following sections.

## Fucosylation in digestive inflammatory diseases

Through genome-wide association studies (GWAS), genetic mutations of some FUTs were discovered to be associated with susceptibility to inflammatory diseases such as inflammatory bowel disease and chronic pancreatitis. Among them, the non-secretor genotype of FUT2, which does not express ABO antigens on the mucosa, is typical.^25^^,^^26^ The mechanisms probably involve intercellular interaction mediated by fucosylated glycans on the cell surface, especially the host–microbe interaction and crosstalk between epithelial cells and the immune system. Interestingly, fucosylation influences the inflammation process, and in turn, inflammation may regulate the epithelial fucosylation level.^27^

## Fucosylation in inflammatory bowel disease

### Clinical association of FUT mutations with inflammatory bowel disease

A decade ago, the association of nonsense mutation of FUT2 with Crohn's disease (CD) was found. McGovern et al found that there was a strong association between the FUT2 single nucleotide polymorphisms (SNPs) rs504963, rs676388, rs485186, rs602662, and rs601338 (FUT2 non-serector status) and CD in Caucasians.^25^ However, another study revealed different results in colonic CD in Japanese, indicating that the frequency of secretors in the colonic CD patients was higher than that in healthy controls and the expression of blood type antigens was a specific feature of colonic CD.^28^ The results might vary among populations. A study in Finnish people found no association between FUT2 rs601338 and CD.^29^ On the contrary, Battat et al found that rs601338 mutation may contribute to the favorable clinical course in CD, and they speculated that FUT2 mutations may be a compensatory protective mechanism.^30^ The proportion of non-secretors was higher among Belgian CD subjects but no such difference was found in the Italian subjects, compared with healthy controls.^31^ Studies in Asians indicated that FUT2 gene polymorphisms were associated with the susceptibility to CD and the secretor status served as protective factors against CD.^32^^,^^33^ Although the above studies suggested no correlation between FUT2 mutants and ulcerative colitis, a study found that rs281377, rs1047781, and rs601338 were associated with ulcerative colitis in Han and Uyghur populations of Chinese.^34^ Therefore, studies involving different populations with larger sample sizes are required to confirm these conclusions. In addition, the FUT3 SNPs may also related to an increased risk of CD and distal colitis in ulcerative colitis.^35^^,^^36^

### Mechanisms of aberrant fucosylation-related intestinal inflammation

**Figure 2:**
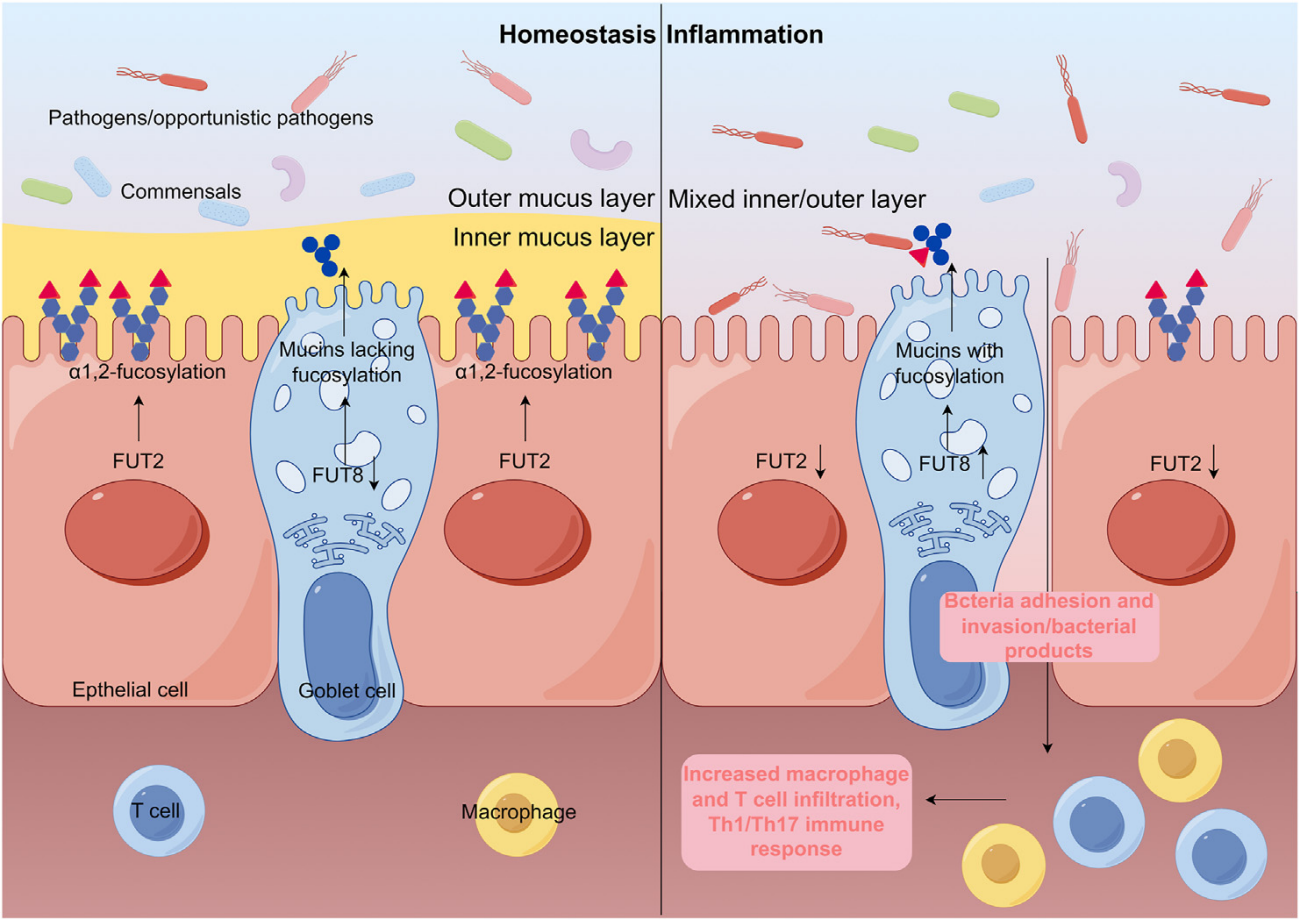
The roles of FUT2 and FUT8 in intestinal homeostasis and inflammation. FUT2-mediated α1, 2-fucosylation on IECs functioning at the interface of host–microbe interaction. The commensals adhesion prevents the invasion of pathogens. FUT2 expression is reduced in inflammation, leading to more colonization of pathogens and more severe barrier dysfunction. The increased FUT8 expression promotes the hypersecretion of fucosylated mucins for bacterial binding and invasion into the intestinal epithelium. Bacterial invasion and bacterial products consequently promote the infiltration of inflammatory macrophages and T cells, aggravating the inflammation. FUT2/8, fucosyltransferase 2/8; IECs, intestinal epithelial cells.

The primary mechanism of fucosylation-related inflammation is host–microbe interaction (Fig. 2). In non-secretor individuals, the abundance of the gut microbiome is lower than in secretors, and the composition and metabolic function of the gut microbiome at the intestinal mucosal surface are significantly different from those of secretors. These alterations are accompanied by sub-clinical inflammation and increased risk of CD.^37^ A recent study revealed that the commensal bacteria-binding fucosylated glycoconjugates decreased in FUT2 loss-of-function mutant individuals, which reduced abundances of adherent bacteria such as *Escherichia* and thereby led to the overgrowth of bacteria which could activate inflammatory T cells.^38^ Experiments on gene knockout mice also demonstrated more severe inflammation in the fucosylation-deficient intestine and more colonization of pathogens such as *Citrobacter rodentium*, *Salmonella typhimurium*, and indigenous *Escherichia coli*.^39^, ^40^, ^41^ Moreover, it was demonstrated that structural and functional changes of gut bacteria in FUT2 knockout mice promoted the generation of lysophosphatidylcholine, which could damage the tight junction and facilitate the production of inflammatory factors.^42^ POFUT1 was also a protective factor for the homeostasis of intestinal epithelium and gut microbes, as it is required for Notch ligand binding. POFUT1 deletion was found to induce Notch inactivation, accumulation of intestinal secretory cell lineages, mucus hypersecretion, and crypt hyperplasia, which altered mucus-associated gut microbiota and contributed to the development of enterocolitis in mice.^43^ On the contrary, it was suggested that the expression of FUT8 in inflamed distal colon tissues of individuals with ulcerative colitis was higher than in healthy controls, which promoted hypersecretion of mucins and bacterial binding and invasion into the intestinal epithelium.^44^

Since FUT2 regulates the composition of gut microbes, it is expected that the frequency of gut microbiota-related T cells changes when there is a FUT2 mutation. In non-secretor individuals, the abundance of CD8 T cells inducing *Alistipe* and *Phascolarctobacterium*, as well as Th17 cells inducing *Erysipelotrichaceae* UCG-003 was increased, which may contribute to the development of inflammatory bowel disease.^38^ Another study discovered the association between wild-type FUT2 and DP8α regulatory T cells induced by symbiont *Faecalibacterium prausnitzii* in rotavirus gastroenteritis, which plays an anti-inflammatory role.^45^

Core fucosylation is the main type of fucosylation that is directly involved in immune regulation in inflammatory bowel disease. The levels of core fucosylation were elevated on T cells derived from inflamed mucosa. Meanwhile, T cell receptor complexes on T cells in FUT8 deficiency mice could not properly engage in signaling transduction, thereby reducing the severity of colitis.^7^ Similarly, the loss of core fucosylation in macrophages impaired CD14-dependent TLR4 and TLR2 signaling, suppressed the secretion of inflammatory cytokines, and protected mice from dextran sulphate sodium-induced colitis.^46^ Furthermore, FUT7 participated in immune regulation in colitis. The deletion of FUT7 was shown to reduce the recruitment of leukocytes and the severity of dextran sulphate sodium-induced colitis, mainly by regulating the combination of selectins and their ligands.^47^

### Potential fucosylation-related therapy approaches in inflammatory bowel disease

The impaired fucosylation of the intestinal epithelium is associated with barrier damage and microbiome dysbiosis, and thus increasing the exogenous fucosylation pathway might be helpful. In the mouse model of colitis, an oral supplement of l-fucose to activate the salvage synthetic pathway of fucosylation protects the intestinal epithelial cells from inflammatory injury. Our previous study found that l-fucose administration in the dextran sulphate sodium-induced colitis mouse model significantly reduced the severity of inflammation and mortality of mice. Exogenous l-fucose increased the expression of metabolic enzymes in salvage pathway, such as fucose kinase and GDP-fucose-pyrophosphorylase in intestinal epithelial cells, and enhanced the global fucosylation in intestinal epithelial cells and intestinal stem cells in mice. Moreover, FUT2 knockout impaired the protective effects of l-fucose and inhibited the fucosylation, which disrupted the unfolded protein response and induced excessive endoplasmic reticulum stress in intestinal stem cells. These results indicate the significant role of FUT2 in the fucose metabolism of the intestinal epithelium.^48^^,^^49^ Also, l-fucose can alleviate the excessive accumulation of intestinal bile acid (such as Tauro-β-muricholic acid) and restore the compromised regulation of hepatic bile acid synthesis by regulating the bacteria structure, especially by increasing the abundance of *Lactobacillus*, thereby ameliorating the impairment of intestinal epithelium in dextran sulphate sodium-induced colitis.^50^

## Fucosylation in chronic gastritis

A lectin microarray study for glycoprofiling also demonstrated that the fucosylation level of tissue and serum glycoproteins isolated from gastritis patients was higher than that of those from healthy controls, suggesting the potential roles of fucosylated proteins as biomarkers for chronic gastritis.^51^

Studies have described the significant role of FUTs in *H. pylori* infection, which is one of the most important risk factors for gastritis and GC.^52^ The fucosylated glycans on the gastric mucosa are essential for the adhesion of *H. pylori* and subsequent colonization and infection, and this aspect has been well elaborated in recent reviews.^10^^,^^53^ *H. pylori* infection could, in turn, promote the expression of FUTs and fucosylated glycans in gastric epithelial cells, especially FUT1/2-mediated α1, 2-fucosylation,^54^ indicating the potential role of fucosylation in the subsequent pathogenesis of *H. pylori* infection, which demands further research to disclose the mechanisms. In addition, since FUTs may mediate leukocyte recruitment in gastric epithelium injury, further studies concerned with fucosylation-related immune mechanisms in gastritis may be meaningful.^55^

## Fucosylation in pancreatitis

The mutation of FUT2 influences the development of pancreatitis. GWAS demonstrated that FUT2 non-secretor status was significantly associated with high lipase levels in asymptomatic subjects, and increased the risk for both idiopathic and alcohol-related chronic pancreatitis but not acute pancreatitis.^26^ Some researchers hypothesize that this might be because the FUT2 mutation affects the structures of secretory proteins and subsequently makes acinar cells susceptible to injury.^56^ Nevertheless, it is proposed that further research with a larger sample size that takes environmental factors into account is necessary to validate these results.^57^

Fucosylated glycans have been found to be a biomarker of autoimmune pancreatitis in recent years. The fucosylation of immunoglobulin G1 (IgG1) and IgG4 was higher in the serum of patients with autoimmune pancreatitis than that of pancreatic ductal adenocarcinoma (PDAC), which may differentiate PDAC from autoimmune pancreatitis with high accuracy in differential diagnosis.^58^^,^^59^

In mechanism studies, it was discovered that FUT3 was necessary for the production of CA19-9, one of the most used tumor markers. An increase in CA19-9 was found to lead to acute and chronic pancreatitis, and it can serve as a therapeutic target in pancreatitis. Transduction of human FUT3 and β3GALT5 (beta-1,3-galactosyltransferase 5) genes in mouse cells largely recapitulates the human CA19-9 carrier profile.^60^

## Fucosylation in hepatobiliary inflammatory disease

### Clinical association of FUT mutations with cholangitis

There was little clinical evidence indicating the direct connection between fucosylation-related genes and the progression of hepatic inflammation. FUT2 SNPs were found to be genetic susceptibility factors for primary sclerosing cholangitis, a chronic cholestatic liver disease with progressive inflammation and fibrosis of the biliary tree.^61^ Similar to inflammatory bowel disease, the non-secretor status of FUT2 results in loss of α1,2-fucosylated glycans on bile duct epithelium. This influences the composition of bile bacteria, particularly the significantly elevated *Firmicutes* and decreased *Proteobacteria*, which may contribute to biliary pathology in primary sclerosing cholangitis.

### Fucosylated proteins as biomarkers for hepatobiliary inflammation

Some fucosylated proteins were identified as potential biomarkers. For instance, the increased fucosylation level or different fucosylation forms in α-1-acid glycoprotein 1 (AGP1) and haptoglobin (HPT) could differentiate nonalcoholic steatohepatitis, cirrhosis from healthy controls, or nonalcoholic steatohepatitis from nonalcoholic fatty liver disease. The level of serum fucosylated HPT increased stepwise along with the increased ballooning hepatocyte score, and the prediction model combining fucosylated HPT and Mac-2 binding protein achieved satisfactory efficiency in nonalcoholic steatohepatitis diagnosis and predicting disease severity of nonalcoholic fatty liver disease.^62^^,^^63^

### Mechanisms of aberrant fucosylation-related hepatobiliary inflammation

Intestinal fucosylation was correlated with hepatitis due to the gut–liver axis. However, contrary to the conditions in colitis, a study indicated that intestinal α1, 2-fucosylation contributed to steatohepatitis in mice. FUT2 knockout protected mice from western diet-induced obesity and steatohepatitis by regulating the composition of gut microbiota and restoring bile acid metabolism.^64^ The possible reason might be that the mice used in this study were a whole-gene knockout. Though this might largely simulate the non-secretor status in humans, further studies are necessary to clarify the specific role of intestinal FUT2 in the development of steatohepatitis.

Globally knockout of the FUT2 gene in mice resulted in portosystemic shunting and subsequent microcirculatory disturbances and periductal fibrosis. The serum bile salt levels of these mice were significantly higher than those of wild-type mice and they were more sensitive to bile salt-induced hepatobiliary damage.^65^

In addition, Kuang et al reported the up-regulation of FUT8 in liver fibrosis potentially induced by TGFβ1. Mechanically, the increased FUT8 could inhibit the TGFβ1-induced trans-differentiation and fibrogenic signals in hepatic stellate cells in a negative feedback loop manner, which inhibits the process of fibrogenesis.^66^

### Fucosylation in digestive cancers

Aberrant glycosylation is a hallmark of cancers, contributing to tumor initiation, progression, and metastasis. The alteration of glycan structures on the cell surface is involved in signaling transduction, immune response, and resistance to chemotherapy, serving as an important target for oncology research.^67^ Aberrant fucosylation and its regulation genes have been observed in various kinds of cancers across nearly all systems, influencing the stemness, proliferation, invasion, and metastasis of cancer cells, and the efficacy of immunotherapies.^68^ Currently, the potential of fucosylated glycans and proteins as diagnosis markers and therapeutic targets is quite remarkable. Herein, we mainly discuss the crucial roles of fucosylation and related genes in colorectal cancer (CRC), GC, liver cancer, and pancreatic cancer (PC). The expression, clinical associations, and regulatory effects of FUTs on digestive cancer are summarized in Table 1, Table 2 and will not be elaborated further in the following parts.

**Table 1:** The expression and clinical correlation of FUTs in digestive cancer patients.

Gene	CRC	PC	LC	GC	References
FUT1			↑, advanced stages, and shorter RFS		132,150
FUT2	↓, survival rate				76
FUT3		Poorer outcomes and metastatic rates			113
FUT4	↑, poorer outcomes				90
FUT5			↑ (ICC)		133
FUT6	↑		↑		78,151
FUT7	↑, lymph node status, and AJCC stage		↑		79,152
FUT8	Increases initially and gradually decays	↑	↑		108,134,153
FUT9	↓				86
FUT11		↑, poor outcomes	↑		139
POFUT1	↑, metastatic		↑, immune-suppressive microenvironment	↑, higher T/N classification	102,138,154

In the LC column, the expression alteration is in HCC unless otherwise specified. CRC, colorectal cancer; PC, pancreatic cancer; LC, liver cancer; HCC, hepatocellular carcinoma; GC, gastric cancer; RFS, relapse-free survival; ICC, intrahepatic cholangiocarcinoma.

**Table 2:** The regulatory functions of FUTs in digestive cancer cells.

Gene	CRC	PC	LC	GC	References
FUT1		Cell survival	Stemness		117,132
FUT2	Proliferation and metastasis	Cell survival			75,76,117
FUT3	TRAIL sensitivity	Cell motilities		Proliferation and invasion	60,83,115,155
FUT4	Immune resistance		Multidrug resistance	Migration and invasion	91,156,157
FUT5		Cell motilities	Proliferation and migration (ICC)		115,133
FUT6	TRAIL sensitivity, cell adhesion and metastasis	Cell motilities	PI3K/AKT signaling and multidrug resistance		78,83,115,151,155
FUT7	Metastasis		Proliferation		79,152
FUT8	TRAIL-induced apoptosis and cell–cell adhesion		EMT, multidrug resistance	Proliferation	80,84,134,158
FUT9	Stemness				85
FUT11		Proliferation and migration	Proliferation and mobility	Proliferation, mobility, and tumor growth	139,159,160
POFUT1	Facilitates tumorigenesis and progression		Drives immune evasion	Tumor growth and apoptosis	88,102,138

In the LC column, the effects are on HCC cells unless otherwise specified. CRC, colorectal cancer; PC, pancreatic cancer; LC, liver cancer; HCC, hepatocellular carcinoma; GC, gastric cancer; ICC, intrahepatic cholangiocarcinoma; TRAIL; tumor necrosis factor-related apoptosis-inducing ligand; EMT, epithelial–mesenchymal transition.

## Fucosylation in CRC

### Clinical association of FUT mutations with CRC

Like inflammatory bowel disease, the SNP of FUT2 was correlated to CRC. When combined with long-term or recurrent antibiotic use, the mutation of FUT2 rs281377 was found to be associated with the risk of early-onset colorectal cancer. Long-term or recurrent antibiotic use seemed to confer a relatively greater risk for early-onset adenomas with the rs281377 TT genotype.^69^ Additionally, the deletion mutation of GMDS was detected in some primary CRC tissues and metastatic lesions, which were not in normal tissues.^70^

### Fucosylated proteins as biomarkers for CRC

The HPT is a representative potential biomarker that could be a useful marker for predicting the prognosis of CRC.^71^ The alpha-l-fucosyl residue was found to be higher in serum beta-HPT of colon cancer patients than that of healthy subjects. Further study showed that Lewis-type and core-type fucosylated N-glycans on four potential glycosylation sites Asn184, Asn207, Asn211, and Asn241 of HPT in serum of gastroenterological cancer were increased.^72^

The fucosylated IgG is another potential biomarker. High-throughput ultra-performance liquid chromatography analysis revealed that CRC was associated with an increase in core-fucosylation of neutral glycans and a concurrent decrease of core-fucosylation of sialylated glycans on IgG, which might be important for disease course prediction and therapy choice.^73^^,^^74^

### Regulatory mechanisms of aberrant fucosylation in CRC

**Figure 3:**
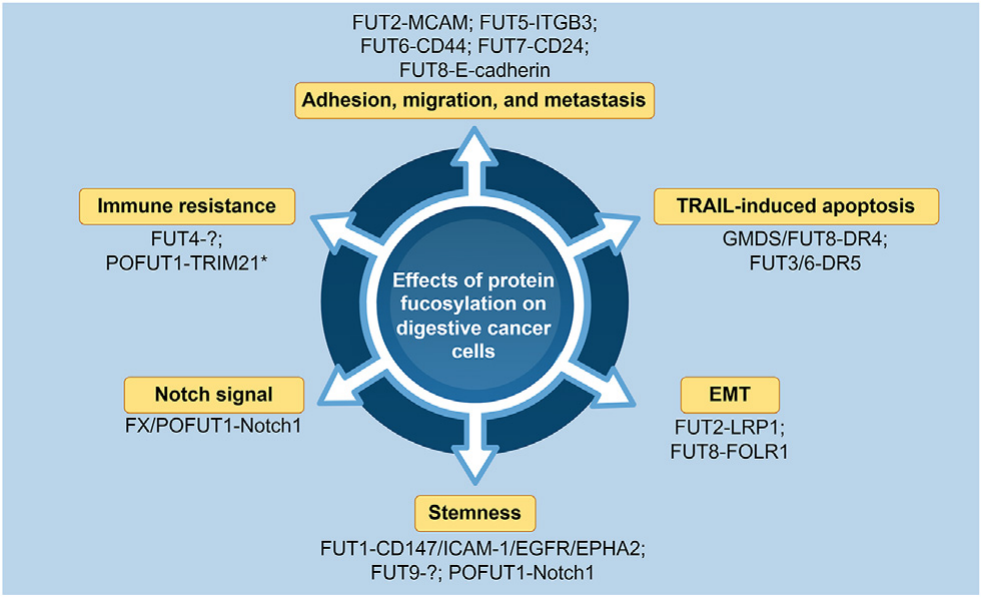
Effects of fucosylation target proteins on digestive cancer cells. FUTs and target proteins that influence the characteristics of digestive cancer cells are shown in the figure. ∗POFUT1 prevents TRIM21-mediated PD-L1 ubiquitination independently of its O-fucosyltransferase activity. FUTs, fucosyltransferases; POFUT1, protein O-fucosyltransferase 1; TRIM21, tripartite motif containing 21; PD-L1, programmed cell death ligand 1.

Since the primary function of FUTs is to mediate the fucosylation of proteins, disorders of FUTs-mediated fucosylation in tumors lead to the dysfunction of these proteins, which might facilitate tumor progression (Fig. 3). Studies discovered that intestinal epithelium-specific Fut2 knockout promotes CRC tumorigenesis in mice and overexpression of FUT2 can suppress the proliferation and metastasis of CRC cells, to a certain extent, by regulating α1,2-fucosylation and the functions of melanoma cell adhesion molecule (MCAM) and low-density lipoprotein receptor-related protein 1 (LRP1), which mediate cell adhesion and cell migration. FUT6 was associated with the cell adhesion and metastasis mediated by E-selectin in CRC cells, which might be attributed to the regulation of α1, 3-fucosylation of E-selectin ligand, CD44, further triggering phosphoinositide 3-kinase (PI3K)/protein kinase B (AKT)/mammalian target of rapamycin (mTOR) pathway.^77^^,^^78^ Overexpression of FUT7 was demonstrated to facilitate metastasis of CRC cells by promoting the carbohydration modification of glycoprotein CD24.^79^ Furthermore, FUT8-mediated core fucosylation regulates the processing of oligosaccharides on E-cadherin and affects the turnover of E-cadherin, subsequently regulating cell–cell adhesion in colon cancer cells.^80^

As promising therapeutic targets for anti-cancer treatment, tumor necrosis factor-related apoptosis-inducing ligand (TRAIL) and activators of its receptor (death receptor 4/5, DR4/5) have attracted considerable attention. Fucosylation is closely associated with TRAIL sensitivity by modifying DRs. GMDS deficiency in colon cancer cells leads to resistance to TRAIL-induced apoptosis by inhibiting the transition from a primary death-inducing signaling complex (DISC) to a secondary complex II, thereby inducing tumor development and evading natural killer cell-mediated tumor immune surveillance.^81^^,^^82^ Colon cancer cells with low FUT3/6 expression were insensitive to DR5-mediated apoptosis, while overexpression of FUT3/6 restored the TRAIL sensitivity through increasing DISC and caspase 8 activation.^83^ Conversely, the knockdown of FUT8 significantly enhanced TRAIL-induced apoptosis and the effects of chemotherapeutic agents in TRAIL-sensitive colon cancer cells but had no impact on TRAIL-resistant cells.^84^^,^^85^ However, the knockdown of FUT9 promoted proliferation and migration in monolayers but inhibited the stemness of colon cancer cells, which might be because the inhibition of FUT9 attenuates tumor-initiating cells (TICs) but promotes bulk tumor cells.^86^

As a classical fucosylated protein, Notch is critical for regulating tumor progress and represents a potential therapeutic target. It was discovered that FX-deleted mice exhibited intestinal dysplasia, and up to 40% of them would develop adenocarcinoma in the colon and cecum. This might be associated with the loss of Notch activation and the decreased expression of its target Hes family BHLH transcription factor 1 (HES1).^41^ The significant role of POFUT1 as a candidate driver of tumor progression in CRC has garnered increasing attention in recent years. POFUT1 expression was correlated with the enrichment of Notch signaling. Deletion of POFUT1 in mice leads to inhibition of Notch1 signaling in CRC.^87^^,^^88^ In addition, POFUT1 and PLAGL2 were co-expressed under the control of an evolutionarily conserved bidirectional promoter, which was disrupted and overexpressed in CRC, thereby facilitating colorectal tumorigenesis by maintaining the stemness of cancer stem cells through the Wnt and Notch pathways.^89^

The roles of FUTs in immune regulation in digestive cancer are little studied. FUT4 was overexpressed in most CRC tissues and was correlated with lower intra-tumoral CD3^+^ and CD8^+^ T cells.^90^ FUT4 could also direct CD15-mediated immune resistance in CRC and may increase the risk of disease progression in response to therapy.^91^

### Potential fucosylation-related therapy approaches in CRC

A recent study found that in HCT116 colon cancer cells, l-fucose treatment simultaneously inhibited cell growth and enhanced the α1,2 and α1,3/6- fucosylation. Intriguingly, the author found that exogenous fucose here did not increase fucosylation by influencing the *de novo* or salvage pathways, but by promoting the biosynthesis of serine, one of the fundamental amino acids for fucosylation modification.^92^ Another study suggested the remarkable role of fucosylation in activating DR5. Low FUT3/6-expressing cells DLD-1 and HCT 116 were insensitive to DR5. Nevertheless, pre-treatment of l-fucose significantly promoted DR5-mediated cell death in these cells, which was similar to the results of overexpression of FUT3/6^83^. Though inhibitory effects of l-fucose in CRC cells were found in *in vitro* studies, its *in vivo* effects needed further studies.

2-fluoro-fucose (2FF), the most widely used fucose analog for fucosylation inhibition, can be converted to GDP forms and deplete the normal GDP-fucose within cells, thereby inhibiting fucosylation.^93^ 2FF treatment showed promoting effects in colon cancer cells. After being pre-treated with 2FF, DLD-1 cells became insensitive to TRAIL-induced apoptosis. In another study, 2FF increased the proliferation, migration, and invasion of SW480 cells.^75^^,^^83^ Compared with 2FF, other more efficient analogs have been developed in recent years. For example, 6,6-difluoro-l-fucose and 6,6,6-trifluoro-l-fucose displayed potent inhibitory effects against colon cancer cells *in vitro*, and the authors suggested that the most likely target is GMDS.^94^ These results further demonstrated the variable role of fucosylation in different conditions, which might limit the clinical application of substrate fucosylation inhibitors.

Inhibiting the fucosylation without specificity may cause many side effects *in vivo*. Thus, selected FUT inhibitors are necessary. A recent study reported a novel FUT8 selected inhibitor, FDW028, which exerts potent inhibitory activities on the malignance of CRC cells and shows comparable anti-tumor effects with 5- fluorouracil in CRC xenografts models. Further experiments indicated that FDW028 treatment defucosylated B7–H3, a key immune checkpoint molecule, promoted lysosomal degradation, and inhibited AKT/mTOR signaling.^95^

In addition to selected FUT inhibitors, targeting fucosylated glycoproteins with antibodies might be another specific strategy. In a recent study, the authors found the expression of heavily fucosylated glycans was higher in CRC tissues and cells than that in normal ones. They then developed an anti-heavily fucosylated glycan monoclonal antibody to explore the anti-tumor effects and discovered that the antibody exhibited dose-dependent cytotoxic effects on CRC both *in vitro* and *in vivo*, especially when combined with chemotherapy.^96^

## Fucosylation in GC

### Clinical association of fucosylation-related gene mutations with GC

The variation of FUT loci was found to be correlated with subtype-specific GC risk in a European population. Diffuse-type GC was associated with FUT5 rs10426709, while intestinal-type GC was related to FUT2 rs10415215 and rs281380, FUT3 rs11673407, and FUT6 rs778809. FUT2 rs281380 was also associated with non-cardia location. The permutation test further validated these FUT loci as determinants of subtype-specific GC risk, and the haplotype analysis indicated that haplotypes in these FUTs were associated with subtype-specific and overall GC risk.^97^

The positive immunostaining of TSTA3 was related to lower histological grades I and II, and the mRNA expression of TSTA3 was associated with histological grade in well and moderately differentiated GC. The expression of alpha-l-fucosidase 2 (FUCA2) was significantly elevated in GC tissues compared with non-tumoral tissues and was associated with TNM III and IV and advanced histological grade tumor states. Both TSTA3 and FUCA2 were correlated to intestinal-type GC classification.^98^

### Fucosylated proteins as biomarkers for GC

IgG is a representative glycan non-invasive biomarker of GC. Although only a minor change of fucosylation of the total IgG Fc fragment in GC patients was discovered, Fc fucosylation was significantly increased in tumor stages II and III, indicating the potential relation to tumor progression.^99^ Another study found IgG fucosylation was increased in GC with peritoneal metastasis compared with advanced GC.^100^

The core fucosylation level and fucosyltransferase in serum and tissues of GC patients were found to be significantly reduced but were reversed after curative surgery. In this study, the author constructed a diagnostic model to distinguish GC from the control based on the profile, and it had a superior diagnostic performance than CEA, CA19-9, CA125, or CA72-4.^101^

### Regulatory mechanisms of aberrant fucosylation in GC

In addition to the role of fucosylation in the *H. pylori* infection, POFUT1 showed effects on GC development. A study found that the overexpression of POFUT1 promotes the proliferation, migration, and invasion of GC cells, accelerates GC growth *in vivo*, and inhibits apoptosis, which could be reversed by Notch inhibitor. Further experiments indicated that the Notch/Wnt dual signaling pathways regulated the carcinogenesis of POFUT1, as cells overexpressing the Notch1 intracellular domain (NICD1) exhibited higher cell growth compared with the negative control while silencing of β-catenin inhibited cell growth in these cells. This might be because POFUT1 governs the parafibromin-NICD1-β-catenin heterotrimeric complex.^102^ Interestingly, a recent study identified a novel circular RNA circPOFUT1 encoded by POFUT1, which could enhance malignant phenotypes and autophagy-associated chemoresistance in GC mice independent of affecting POFUT1 expression. circPOFUT1 was demonstrated to sequestrate miR-488-3p, which directly targets PLAG1 to reduce ATG12 expression, thereby activating the PLAG1-ATG12 axis-mediated autophagy.^103^

### Potential fucosylation-related therapy approaches in GC

A recent study revealed the potential function of fucosylation inhibitors in GC therapy. It was reported that fucosylation is indispensable for the anti-tumor activities of KIAA1324, a transmembrane tumor suppressor protein and favorable prognosis marker in GC. In MNK28 GC cells, the inhibition of fucosylation by 2F-peracetyl-fucose abolished KIAA1324-mediated cell growth inhibition and decreased KIAA1324-mediated apoptosis in a dose-dependent manner. Mechanistically, the loss of fucosylation might influence the stability and localization of KIAA1324 and is necessary for KIAA1324 to block the GRP78 (78 kDa glucose-regulated protein)-caspase-7 interaction, which inhibits tumor formation by inducing apoptosis.^104^

## Fucosylation in PDAC

### Clinical association of fucosylation with PDAC

ABO blood group is one of the risk factors of PDAC, the primary subtype of PC,^105^ which is associated with FUT mutation. However, the results have been somewhat inconsistent thus far. The risk of PC is found to increase in subjects with non-O ABO blood group alleles. Although a previous study revealed the association between the ABO blood group and pancreatic cancer was not influenced by the secretor status, a recent study suggested that the increased PC risk in non-O ABO blood groups was more obvious in the secretor phenotype than in non-secretors, and there was no interaction between blood groups and Lewis antigens.^106^^,^^107^

Fucosylation was also associated with pancreatic intraductal papillary mucinous neoplasm. There was a sequential increased aleuria aurantia lectin (AAL) and aspergillus oryzae l-fucose-specific lectin (AOL)-binding fucosylated-glycans, as well as the expression of FUT8 in tissues ranging from normal pancreatic duct to adenoma and carcinoma, indicating the potential correlation of fucosylation with the malignant transformation of intraductal papillary mucinous neoplasm.^108^

### Fucosylated proteins as biomarkers for PDAC

Serum fucosylated glycans were increased in PDAC patients, and tri-antennary fucosylated glycans underwent significant temporal changes. This may help the early detection of the cancer with a specificity of 92%, a sensitivity of 49%, and an accuracy of 90%.^109^ Increased α1, 3-fucosylation, sLe^x^, and core fucosylation of AGP, increased core fucosylation of HPT, and decreased fucosylation of IgG1 were identified in the serum of PC patients compared with that of healthy controls.^110^^,^^111^ The serum fucosylated AGP was also positively correlated with the malignant potential of intraductal papillary mucinous neoplasm.^112^

### Regulatory mechanisms of aberrant fucosylation in PDAC

It was discovered that Lewis antigen-negative PC patients exhibited poorer prognoses and higher metastatic rates, and Lewis-negative cells (CaPan-1, MiaPaCa-2, and Panc-1) possessed higher proliferation and migration ability than Lewis-positive cells (BxPC-3, SU8686, and SW1990).^113^ Other studies, however, found that knockdown of FUT3 (which participates in regulating Lewis antigens) inhibited the proliferation, migration, tumorigenesis, and epithelial–mesenchymal transition of CaPan-1, and FUT3, 5, 6 mediated fucosylation enhanced cell motilities in metastatic pancreatic cancer lines CaPan-1 and Su.86.86.^114^^,^^115^ A likely reason is that FUT3 is not the only enzyme to regulate Lewis antigens. Additionally, FUT3-mediated production of sialyl-Lewis^A^ (also known as CA19-9, the most widely used serological marker of PC) could cooperate with Kras^G12D^ oncogene to generate aggressive PC.^60^ Thus, combined CA19-9 levels and gene tests to detect FUT variants can significantly improve the diagnostic accuracy of PDAC.^116^

Oxygen deprivation is a common characteristic of solid tumors. Under the hypoxia condition, FUT11 was identified as a crucial hypoxia-associated gene regulated by hypoxia-inducible factor 1α (HIF1α), which promotes PC cell survival under oxygen and glucose deprivation. HIF1α binds to the promoter of FUT11 and increases its transcription, while another study identified HIF1α as one of the suppressors of FUT1/2 expression in PDAC.^117^

## Fucosylation in hepatobiliary tumor

### Clinical association of FUT mutations with hepatobiliary tumor

The genetic risk of FUT SNPs in liver cancer was also identified. Compared with the wild-type genotype, the mutant of FUT2 rs1047781 exhibited a significant association with the clinical stage and tumor size of hepatocellular carcinoma (HCC).^118^ In addition, it was discovered in patients with primary sclerosing cholangitis that the FUT2 variant rs601338 correlated with CEA levels, particularly in patients genetically incapable of expressing CA19-9. The genotypes of FUT2 and FUT3 could also determine the cut-off values of CA19-9 for the detection of cholangiocarcinoma in patients with primary sclerosing cholangitis. Combining CEA, CA19-9, and FUT genotyping could enhance the early detection of biliary malignancy.^119^^,^^120^

### Fucosylated proteins as biomarkers for hepatobiliary tumor

To date, numerous fucosylated proteins have been identified as biomarkers of liver cancers through liquid chromatography-mass spectrometry analysis, and the alteration of these proteins might be ascribed to the aberrant expression of FUTs.^121^ Fucosylated HPT is the most extensively studied fucosylated biomarker in liver cancer. There exists a unique pattern of core and antennary fucosylation in HPT of HCC patients, and the degree of fucosylation was elevated in HCC patients compared with healthy controls and cirrhosis, thereby making fucosylated HPT and the fucosylated HPT/HPT ratio potential markers for the early detection of HCC and the discrimination of HCC from cirrhosis.^122^, ^123^, ^124^, ^125^ The serum α-fetoprotein (AFP) concentration is the most used marker of HCC. The degree of fucosylation of AFP was proved to be beneficial for distinguishing HCC from benign liver diseases when the AFP concentration was lower than 1000 ng/mL, which is insufficient for this purpose.^126^^,^^127^

Regarding cholangiocarcinoma, fucosylated fetuin-A may be valuable in differentiating cholangiocarcinoma from primary sclerosing cholangitis and the surveillance of individuals at risk of cholangiocarcinoma.^128^ Combining the alterations of fucosylated proteins with other N-linked glycans typically provides a more accurate diagnosis or prediction result.^63^^,^^129^

### Regulatory mechanisms of aberrant fucosylation in hepatobiliary tumor

GDP-fucose-related proteins were associated with liver cancers. FX and GDP-fucose transmembrane transporter (solute carrier family 35 member C1/SLC35C1) were discovered to contribute to the aberrant fucosylation in intrahepatic cholangiocarcinoma. Silencing of FX and SLC35C1 inhibits Notch and EGFR/nuclear factor kappa B (NF-κB) signaling, thereby suppressing the growth and migration of intrahepatic cholangiocarcinoma cells. Furthermore, inhibition of fucosylation suppresses the growth of intrahepatic cholangiocarcinoma cells in the chick chorioallantoic membrane assay.^130^ A systematic review also identified GMDS and FX as the contributors to elevated core fucosylation in HCC.^131^

In HCC, the glucose deficiency microenvironment enhanced the expression of FUT1 by promoting the binding of activating transcription factor 4 (ATF4) to the FUT1 promoter. FUT1 targets the fucosylation of CD147, intercellular adhesion molecule 1 (ICAM-1), EGFR, and EPH receptor A2 (EPHA2), consequently converging on the down-regulation of AKT/mTOR/eukaryotic translation initiation factor 4E-binding protein 1 (4EBP1) signaling to enhance cancer stemness.^132^

FUT5 was overexpressed in intrahepatic cholangiocarcinoma tissues compared with corresponding adjacent nontumor tissues, which promotes the proliferation and migration of intrahepatic cholangiocarcinoma cells by mediating the fucosylation of versican and β3 integrin, which are involved in the interaction of cells with the extracellular matrix to exert precancer effects.^133^

Core fucosylation is increased in HCC individuals. The loss of FUT8 can inhibit the tumorigenesis of HCC in a chemical-induced HCC mouse model and xenograft tumor model.^134^ Some non-coding RNAs were discovered to regulate the expression of FUT8 in HCC. circRNA cFUT8 was demonstrated to facilitate HCC cell development and sustain malignant potential by positively modulating the expression of FUT8 via binding to free miR-548c and suppressing the miR-548c/FUT8 regulatory axis.^135^ Long non-coding RNA HOTAIR activated STAT3, a regulator of FUT8, to promote FUT8 expression, while FUT8 could promote the core-fucosylation of Hsp90, which stabilized the mucin 1 (MUC1) binding to the downstream p-STAT3. This feedback loop modulated the progression of HCC.^136^ Folate receptor α (FOLR1) was identified as one of the downstream targets of FUT8. The core-fucosylation of FOLR1 was up-regulated during the epithelial–mesenchymal transition process of the HCC cells and enhanced its folate uptake. Meanwhile, the knockout of FUT8 inhibits the fucosylation of FOLR1 and partially blocks the epithelial–mesenchymal transition.^137^

A recent study identified POFUT1 as an essential driver of immune evasion in HCC. POFUT1 up-regulates the stability of programmed cell death ligand 1 (PD-L1) by preventing tripartite motif containing 21 (TRIM21)-mediated PD-L1 ubiquitination and inhibiting its degradation, thereby inhibiting CD8^+^ T-cell infiltration and reducing the production of cytotoxic molecules. Moreover, the inhibition of POFUT1 enhances the efficacy of anti-PD-1 therapy in HCC in mouse models.^138^

The silencing of FUT11 restrains the proliferation and mobility of HCC cells under both normoxia and hypoxia,^139^ which is similar to the results in PC, suggesting that there are similarities in the effects of FUTs in different kinds of cancers.

Furthermore, FUTs might play certain roles in hepatitis virus-related HCC. Hepatitis B virus (HBV) infection and the overexpression of HBV X protein in HCC cells down-regulated microRNA-15b and consequently induced FUT2-mediated Globo H expression, which was demonstrated to promote HCC cell proliferation.^140^ Likewise, hepatitis C virus infection could induce FUT8 overexpression in the HCC cell line and cause chemotherapy resistance by stimulating the expression of the drug-resistant proteins P-glycoprotein (P-gp) and multidrug resistance-related protein 1 (MRP1).^141^

### Potential fucosylation-related therapy approaches in hepatobiliary tumor

Fucosylation inhibitors exert inhibitory effects on liver cancer cells. Researchers discovered that 2FF treatment suppressed the proliferation of HepG2 cells by inhibiting the core fucosylation of EGFR and integrin β1, which affects their activation and suppresses downstream signaling.^142^ 6-alkynyl-fucose has been identified as a general inhibitor of endogenous fucosylation that primarily targets FX. It was found to prevent the migration and invasion of hepatoma cells and was more effective than 2FF.^143^ Additionally, 6-alkynyl-fucose inhibited fucosylation in intrahepatic cholangiocarcinoma cells and consequently suppressed their tumorous characteristics, and the Notch and EGFR activity.^130^

## Conclusions

It is indisputable that fucosylation plays an important role in the parthenogenesis and development of digestive inflammatory and tumorous diseases encompassing nearly all organs. In this review, we tried to illuminate the clinical association, biomarkers, molecular mechanism, and therapeutic potential related to fucosylation based on up-to-date evidence. In fact, there remain lots of fields that require future investigations.

On the one hand, the mechanism research is still inadequate. Although the altered expression of many FUTs has been identified to be correlated with the proliferation, metastasis, and drug resistance of cancer cells, more studies are necessary to explore the underlying working mechanism. Besides, the role of fucosylation in inflammation-cancer transformation and its regulatory effects in cancer stem cells can be further studied.

On the other hand, the clinical application of fucosylation-related strategies in the diagnosis and treatment of diseases is still limited. Although many fucosylated biomarkers were identified, the cutoff and the specific fucosylated sites for the diagnosis of different kinds of diseases are not well-defined. Furthermore, although some fucosylated glycans were found to be altered in lesions, how these aberrant fucosylated glycans influence disease progression still demands further study.^144^ Our group devised a diagnosis strategy employing confocal laser endomicroscopy to detect UEA-I-FITC stained α1, 2-fucosylation on the surface of the colon for CRC detection since the fluorescence intensity ratio was lower in tumor tissue. This presented a sensitivity of 95.6% and a specificity of 97.7% and improved the diagnostic efficacy of confocal laser endomicroscopy.^145^ This may offer ideas for the clinical diagnostic implication of fucosylation. Regarding therapy strategies, if the aim is to increase the fucosylation level of the digestive tract, l-fucose could become a potential candidate. A recent study discovered dietary l-fucose increased anti-tumor immunity and enhanced immune checkpoint blockade responses in melanoma by inducing fucosylation and enrichment of HLA-DRB1 in a mouse model.^146^ The therapeutic potential of fucose in digestive cancers through immune activation could be further studied, which might improve the application of fucose in digestive diseases. Also, it should be suggested that there are still unclearness and controversy in the therapeutic effects and mechanisms of fucose administration, which limits the further application of fucose in clinical experiments. Microbe modulation might be another candidate for fucosylation regulation. *Bacteroides fragilis*, a kind of commensal bacteria showed potential benefits for health because it induces α1, 2-fucosylation in intestinal epithelium.^18^^,^^147^ A recent clinical trial indicated its advantages in alleviating the gastrointestinal toxicity of chemotherapy.^148^ Therefore, future clinical research could explore its therapeutic efficacy on fucosylation deficiency-related digestive disorders. For suppressing fucosylation, most of the experiments of fucosylation inhibitors were performed *in vitro*, and generally inhibiting the fucosylation without specificity might lead to many side effects *in vivo*, since the diverse roles of fucosylation and FUTs in distinct tissues and diseases. A phase I trial in advanced solid tumors showed the preliminary antitumor activity of 2FF. However, the researchers discovered that 2FF was associated with thromboembolic events that led to the termination of the study.^149^ The *in vivo* applications of selected fucosylation inhibitors are still limited. Verification of the effectiveness and safety of novel inhibitors using *in vivo* models and subsequent clinical trials, if possible, might provide new perspectives on the therapeutic strategies for aberrant fucosylation-related diseases.

## CRediT authorship contribution statement

**Caihan Duan:** Writing – original draft, Conceptualization. **Junhao Wu:** Investigation. **Zhe Wang:** Investigation. **Xiaohua Hou:** Supervision, Funding acquisition, Conceptualization. **Chaoqun Han:** Supervision, Funding acquisition.

## Funding

This study was financially supported by the 10.13039/501100001809National Natural Science Foundation of China (No. 92268108, 82170570, 81974062).

## Conflict of interests

The authors declared no competing interests.
